# COVID, Vulnerability, and the Death of Solidarity: “Who Do We *Not* Save?”

**DOI:** 10.1007/s11673-023-10250-x

**Published:** 2023-07-11

**Authors:** J. L. Scully

**Affiliations:** Disability Innovation Institute, University of New South Wales, Sydney, NSW 2066 Australia

**Keywords:** COVID-19, Solidarity, Disability, Vulnerability

## Abstract

Solidarity between more and less vulnerable groups is fundamental to an effective public health response to a global pandemic. Yet in the case of COVID-19, a focus on deciding who can and who cannot be protected from harm has shaped the pandemic experience and continues to determine the post-pandemic trajectory of life with SARS-CoV-2. In this paper I discuss how this has affected our understanding and acceptance of solidarity.

Towards the end of the first year of the COVID-19 pandemic, U.K. government advisor Dominic Cummings published a photo of a whiteboard that had been used in a brainstorming meeting held in the Prime Minister’s office at 10 Downing Street on 13 March 2020. Near the bottom of the whiteboard someone has scribbled a final question: Who do we *not* save?^1^

This question, posed this way, has crucially shaped the pandemic response of many countries. In doing so it has also shaped the experiences of those living (and dying) in the pandemic and continues to determine the post-pandemic trajectory of life with COVID. In this paper I discuss the effect this has had on our experience of solidarity and on the collective trust that societies will act responsibly towards their more vulnerable members.

## Drawing Lines

COVID-19 was and is a disease affecting every nation in the world.^2^ Nevertheless, the first lines of differentiation across populations in countries including the United Kingdom, United States, and Australia began to appear even as the political discourse continued to be of shared risk: *we’re all in this together*. Observing different jurisdictions’ pandemic responses is instructive in what they say about societies’ readiness to sort people and groups into those that matter and those that don’t. It will take extensive future research to map these processes of pandemic social sorting and to understand how the specifics of history, geography, politics, and economics organized them differently across different countries and cultures. This discussion focuses primarily on Australia, although much of it is more widely applicable.

Living in Sydney I heard the first vague news of a novel viral disease, symbolically enough, on the very last day of 2019. Less than three months later much of the world was in lockdown. I was working from home and, along with everyone else, trying to predict whether this was something that we would eventually look back on as a blip in the course of our lives or whether it was, in fact, the zombie apocalypse.

So little was first known about the disease course or the virus’ natural history that there was a real sense of potential vulnerability as universal. Although the pandemic was initially a distant threat heading towards the southern hemisphere from the other side of the world, by January 2020 the Australian government had closed the country’s borders in an attempt to keep that threat at bay: to protect the vulnerability of the entire continent. But despite these efforts the first cases of COVID in Australia were reported on January 25, 2020. The Federal government initially pursued a zero COVID policy aiming at total suppression; pandemic measures came into force, including social distancing, masking, working from home, the closure of social venues, and several statewide or more localized lockdowns. Interstate border closures also began in March 2020. These measures kept infection rates low relative to the rest of the world.

Initially there was a strong *internal* solidarity with all other Australians (including those now trapped outside the country by closed borders), fostered by targeted public messaging.^3^ A similar political rhetoric promoting national unity was mobilized in many other countries during the first year of the pandemic. Quite soon, however, the consistency of this message began to fragment with evidence that the biological impact of COVID is not uniform on all those who could theoretically be infected. On average the acute disease is least harmful to children and healthy, relatively young adults; it has significantly greater morbidity and mortality in older people and presents higher risk for those with a range of existing health conditions that include heart and lung disease, compromised immunity, diabetes, and hypertension. These interactions between COVID and what were generally termed underlying conditions provide a partial explanation for the overrepresentation of certain groups of people in the statistics for both infection and death. But the reasons why men (for example), or minority ethnic groups, or Indigenous communities, have more infections and severe disease are not straightforwardly physiological. Also in play are well characterized social determinants of health: the more socially and economically marginalized a community, the more likely that its members are already experiencing the pathologies that increase vulnerability to COVID. And in addition, the epidemiological data also show that these populations are more likely to face extra barriers to accessing public health information and care or to have jobs that make it impossible for them to work from home or isolate when sick.

Similar differentials both between and within countries were exposed in the development and provision of vaccination. The speed at which effective vaccines against the first viral variants were produced resulted from impressive levels of national and international scientific cooperation. Nevertheless, recent bioethical literature (Silva et al. 2021; Wagner et al. 2021) has criticized the way that global solidarity dropped out of sight in the face of an extensive “vaccine nationalism” in which countries have focused on the interests of their own citizens alone, despite the knowledge that international gaps in effective vaccination—as well as being fundamentally unjust—encourage the generation of new viral strains, ultimately increasing the risk for everyone.

Australia’s early successes in avoiding uncontrolled spread have to an extent been mirrored in its vaccination programme. At March 1, 2023, 97.5 per cent of the population had had at least one vaccination (Australian Government Department of Health and Aged Care 2023). This compares very favourably with the United Kingdom (82 per cent), United States (80 per cent), or Switzerland (70.4 per cent) (Our World in Data 2023). Even so, vaccination rates are still an average of 10 per cent lower in the equivalent proportion of the Indigenous population, for example, and among Australians with disability.

Biological, societal, and political factors interact so that COVID has had distinctly different trajectories in different countries and in different national populations. Because of this the pandemic and the measures to control it have operated to expose, and in many cases exacerbate, the fissures that run through contemporary societies. They reveal who we think matters, and who does not, and how this plays out in terms of vulnerability and solidarity.

## Vulnerability and Solidarity

Vulnerability has an extensive conceptual history in medical ethics and bioethics, starting from the recognition of the vulnerability of individual subjects in research and extending into areas of clinical care and the vulnerability of population groups in public health (see e.g. Rogers 2014). As recent writing has acknowledged, however, theorizing about vulnerability struggles with a constant central ambiguity. Research, clinical, and public health ethics tend to focus on instances of particular vulnerability where “inequalities of power, dependency, capacity, or need render some agents vulnerable to harm or exploitation by others” (Rogers 2014, 6). But there is an alternative perspective in which vulnerability is something that every human being experiences in their lives, an inescapable part of the human condition (Fineman 2008). This central tension between universal and particular vulnerability is still not adequately resolved, despite recent, more nuanced analyses and taxonomies of different forms of vulnerability (e.g. Luna 2019; see also summary in Luna 2023).

Although it has a long track record in political theory, the concept of solidarity is a relative newcomer to the bioethical landscape. Solidarity combines several ideas of connectedness, of responsibilities that extend beyond one’s immediate circle, and of the moral obligation to attend to the needs of others. Some writers emphasize a political form of solidarity as a “relation that unites individuals acting on the basis of some form of commitment to challenge injustice, oppression, social vulnerability, or to otherwise struggle for liberation” (Scholz 2008, 82). Others understand it primarily as a moral demand to stand alongside people who are personally unknown to you but are nonetheless in need of support and protection that you are in a better position to provide: for Guttman et al. (2016, 913), for example, solidarity is the “collective obligation to attend to the needs and welfare of others, in particular the most vulnerable.” Most writers on solidarity emphasize that it is not just a *feeling* of concern for others in distress, but a *practice*—and one that involves a cost or sacrifice that one person pays for the sake of another. Solidarity recognizes that a collective effort is needed to meet the full range of essential human needs and enable people to flourish. To be unable, for some reason, to cover those needs for oneself, to protect oneself from the harm that results from this, and as a result to depend on others for help, is to be vulnerable. Vulnerability and solidarity are therefore inextricably connected.

Solidarity highlights the idea of acting for the good of a group whose individual identities are unknown to us, rather than for people we know. But some definitions also limit the moral requirements for solidarity by placing it within a framework of similarity. For Prainsack and Buyx, solidarity is the “enacted commitment … to assist others with whom a person or persons recognize *similarity in a relevant respect*” (Prainsack and Buyx 2017, 52, italics added), while Guttman et al. consider it to be “based on people’s recognition of similarity of needs” Guttman et al. 2016, 913). The implication is that solidarity with some entails the exclusion of others. It also means that those showing solidarity (or not) must have a fairly clear sense of the point where similarity “in a relevant respect” breaks down. Who stands inside the circle of people with whom we are in solidarity, and who makes that decision?

## Pandemic Solidarity

During the global pandemic, solidarity with others was demonstrated through the actions of individuals complying with the range of measures that governments brought in (Basaure et al. 2021): wearing masks and sanitizing, social distancing, isolation and quarantine, and the various levels of restriction on everyday movement, working from home, and getting vaccinated. In Australia and elsewhere the rationale for people voluntarily accepting limits to their normal freedom was initially a combination of self-interest (this will keep you personally safe from infection) and solidarity (these measures will also protect others more clinically and socially vulnerable than you). Although often reinforced by legal sanctions, these solidaristic measures still depended on the general population’s endorsement and cooperation.

While many countries mobilized resources to counter the economic and other impacts of measures to suppress the pandemic, it would be naïve to think that this was based on an ethos of broad solidarity. The limits to collectivity were most obvious in neoliberal states such as Australia and the United Kingdom but also became apparent in European countries that are generally considered to have a more collectivist orientation. At different points throughout the pandemic there has been a gradual redrawing of lines between people who matter and those who don’t, or at least matter rather less: a stratification that became increasingly visible as the rhetoric of solidarity died down. Writing about Norway, the disability scholar Patrick Kermit said that although “we all experienced a new and collective form of vulnerability, we tacitly choose to overlook that those already vulnerable had to pay a higher price than most others to uphold the—by all means well intended—restrictions” (Patrick Kermit, pers. comm.).

Stratification has affected a wide range of marginalized groups. In Australia these include culturally and linguistically diverse communities, migrants, disabled and older people, and the Indigenous populations. What this has meant for people with disability or chronic illness provides a particularly stark example of the impact of selective solidarity. Even in places with well developed systems of health and social care, pandemic responses were hampered by a lack of knowledge of the distinctive issues that people with disability were likely to face. This ignorance might explain some of the measures that have since been recognized as counterproductive, such as discharging patients with COVID from hospital back to disability care homes, with the inevitable result that infection spread within closed communities. To give one example of differentiated solidarity, a global survey of the impact of the pandemic on people with intellectual disability and their carers reports that “[i]n contrast to the widespread acknowledgement of the burden of COVID-19 on older persons there has been insufficient recognition of the impact of the pandemic on persons with disabilities, especially those who are resident in congregated settings” (Linehan et al. 2022, 28). People with disability worldwide have expressed suspicion that lack of *knowledge* has been compounded by a lack of *interest*, reflecting disablist assumptions and prejudices in which disabled people are routinely categorized as less useful (indeed, potentially burdensome) in crises, and therefore of less value.

To be clear, the disablism here does not take the form of overt hostility or uniform neglect. For example, the Australian government produced a plan for protecting people with disability during COVID by April 2020 (Australian Government Department of Health 2020), a speedier effort than many other countries, while the national vaccination programme initially gave people with disability priority in phase 1a of the rollout. But significantly, early in 2020 a decision was made to prioritize the vaccination of people in aged care over disabled care. Irrespective of the logic behind that decision, the fact that it was made without consulting or informing representative organizations (Disability Royal Commission 2021) reveals something about how people with disability are positioned relative to the rest of Australian society.

Similarly, the terminology of *underlying conditions* in the daily COVID reports in Australia and elsewhere reinforced the image of two clearly separable groups: those with a notable vulnerability to COVID, primarily disabled and older people, and all the rest. Rhetorically constructing the two categories as having clearly demarcated vulnerabilities to COVID made it seem reasonable for the societal response to their deaths to be different as well. While deaths in the first group were sad but not unexpected, those in the second were seen as extraordinary. However much commentators and public health authorities tried later to introduce more nuance—noting for example the significant number of deaths among people under 60, that “underlying conditions” such as hypertension or diabetes are not exactly rare, or that a growing cohort of people survive the acute illness only to become chronically ill—it has proved almost impossible to dislodge a narrative in which the majority of people need not feel directly threatened by the disease.

A combination of better knowledge about how the virus operates, and changes in public health messaging, saw a shift towards an emphasis on the individual calculation of risk. On February 21, 2022 the British Prime Minister made a statement to the House of Commons on “living with COVID”, in which he said that “we can now deal with it in a very different way, moving from government restrictions to personal responsibility” (Prime Minister’s Office 2022). In NSW mandatory masking, along with most other public health restrictions, had ended by February 2022 (NSW Public Health Orders 2022). In Australia and many other countries, the dropping of pandemic measures has been accompanied by the promotion of individual rather than collective responsibility in which whether or not to adopt protective measures is a matter of personal choice. But the problem with invoking personal responsibility is that it only works if people understand what acting responsibly means in the relevant situation and have the capacity to do so. As became apparent, not everyone can afford to buy effective masks and COVID tests if the state does not supply them; not everyone can responsibly self-isolate if there is no protection against the loss of income or their job. In these circumstances the only responsible action left for disabled, elderly, or otherwise vulnerable people was to opt out of society while the rest of the world returned to something it considered normal. The reduction of public health measures down to making responsible decisions about oneself ignores the core of solidarity: the idea of acting in the interests of others even though it comes at personal cost. Individual *responsibility* alone implies that public health measures are aimed at individual *protection* rather than being part of a collective duty to keep the entire community safe.

## An Enduring Impact

As John Lanchester wrote in the *London Review of Books* in December 2021, “COVID is an almost impossible subject to sum up, because we don’t know where we are in the story” (Lanchester 2021). But there are certain observations we can make now that give an indication of how the next few episodes in the story might play out. For bioethics and public health ethics, the COVID-19 pandemic has pushed a re-evaluation of some key values and principles. It has brought into plain view the complex, interwoven, and often highly politicized relationship between clinical bioethics’ focus on the interests of the individual patient, and public health ethics’ concern with the well-being of groups. It has raised questions about the routine invocation of global solidarity in view of high income countries’ consistent disregard of any attempt towards vaccine equity with poorer countries. More broadly, it has also exposed what happens when health policy fails to articulate convincingly that individual flourishing depends on how the community as a whole is doing.

As much as the disease itself, the measures that attempted to contain it caused widespread social division: in a global pandemic, no one feels that they are having it easy. Nevertheless, some people suffered more than others. Neglect of the needs of particular groups more vulnerable than the average has almost certainly destroyed whatever trust those groups had in a wider solidarity. In response, some marginalized groups have come together to work for political change or provide practical support where it is needed. The Australian umbrella group Advocacy for Inclusion (AFI), for example, successfully lobbied for an extension of mandatory masking in high-risk disability settings towards the end of 2022. Grassroots activism like this is often effective and can be a model for interest group advocacy, but it cannot compensate for the observable failures of civic solidarity.

When a totally new virus like SARS-CoV-2 first emerges, the fate of those it infects is largely a matter of biology and luck. But as knowledge accumulates, biology and luck morph into social and political choices. *Who do we not save*? came to mean *who doesn’t matter (enough) to us*? If solidarity is defined as directed concern towards those with whom we feel we have something in common then—unpalatable as it may be to acknowledge—there are people securely embedded in the social mainstream who are not convinced they have anything in common with those who are poor, foreign, Aboriginal, old, or disabled. In philosophical terms, they don’t feel they share the same personhood or the same moral status. So the pressing ethical question for the future is: to what extent is it possible in a state of crisis to keep solidarity’s boundaries of “relevant similarity” porous and expansive? What would life with COVID—and life with the next pandemic, and the one after that—look like if the starting point is not, *who do we not save*? but, *what should we do to ensure we can save everyone*?

## Footnotes

1 Widely reported in the news media in May 2021; see for example Topping, 2021. What the Cummings whiteboard reveals about the COVID response. *The Guardian*, May 26. https://www.theguardian.com/world/2021/may/26/what-the-cummings-whiteboard-reveals-about-the-covid-response

2 In March 2023, Turkmenistan remains the only country with no official reports of COVID cases, although the accuracy of this reporting has been challenged.

3 Many Australians remember ABC television’s regular broadcasting of the Seekers’ song *I Am, You Are, We Are Australian* in an unsubtle but nonetheless emotionally powerful effort to reinforce that solidarity.
